# Controlled generation of 3D vortices in driven atomic Josephson junctions

**DOI:** 10.1073/pnas.2535111123

**Published:** 2026-07-07

**Authors:** Vijay Pal Singh, Ludwig Mathey, Herwig Ott, Luigi Amico

**Affiliations:** ^a^ Quantum Research Center, Technology Innovation Institute, Abu Dhabi, United Arab Emirates; ^b^ Zentrum für Optische Quantentechnologien and Institut für Quantenphysik, Universität Hamburg, Hamburg 22761, Germany; ^c^ The Hamburg Centre for Ultrafast Imaging, Hamburg 22761, Germany; ^d^ Department of Physics, Research Center OPTIMAS, Rheinland-Pfälzische Technische Universität Kaiserslautern-Landau, Kaiserslautern 67663, Germany; ^e^Dipartimento di Fisica e Astronomia, Università di Catania, Catania 95123, Italy; ^f^INFN-Sezione di Catania, Catania 95127, Italy

**Keywords:** ultracold atoms, driven Josephson junctions, Jones-Roberts excitations, topological defects

## Abstract

We propose an ac-driven atomic Josephson junction as a clean and tunable source of three-dimensional (3D) solitary waves in quantum fluids. Depending on the height of the junction barrier, the emitted excitations appear as vortex rings at low velocity or vorticity-free rarefaction pulses near the sound velocity, thus spanning the complete Jones-Roberts family of solitons. The Shapiro-step phenomenon renders the emission deterministic: on the first, second, third Shapiro steps, the junction ejects one, two, and three solitary excitations per drive cycle. This enables controlled generation of single- and multiexcitation configurations, allowing detailed studies of the full crossover between vortex rings and rarefaction pulses and their interaction dynamics. By Shapiro phase locking, multiexcitations are emitted in succession and interact, revealing leapfrogging motion of two and three coaxial rings and their decay via boundary-assisted, sound-mediated processes. This ac-driven protocol establishes a compact and reproducible platform for generating, classifying, and controlling 3D solitonic excitations, paving the way for precision studies of nonlinear vortex dynamics, dissipation, and quantum turbulence in trapped superfluids.

## Significance

Understanding how quantized vortices form, interact, and decay lies at the heart of quantum-fluid dynamics. We introduce a driven atomic Josephson junction as a versatile platform to create and control these excitations with single-cycle precision. By combining deterministic Shapiro steps-locked emission with tunable junction barrier parameters, we effectively realize an on-demand vortex-ring device able to generate excitations from vortex rings to rarefaction pulses, spanning Jones-Roberts family of 3D solitons. This capability transforms the driven junction into a “piston-cylinder” set up for probing nonlinear wave dynamics, vortex interaction, vortex-phonon coupling, and dissipation in three dimensions. Beyond revealing fundamental mechanisms of superfluid turbulence, the approach offers a reproducible route to engineer coherent, topological flow structures for future atomtronic and quantum-fluid technologies.

Quantum vortex dynamics lies at the core of diverse physical phenomena, ranging from turbulence and astrophysical plasmas to emerging quantum technologies (1–3). Superfluid helium (4, 5) and, more recently, ultracold atomic gases (6–14) provide powerful and highly controllable platforms to explore these quantized vortices under well-defined conditions. These systems enable fundamental questions, such as vortex–vortex interactions, stability, and the transition from classical to quantum turbulence, to be investigated with unprecedented accuracy (15). In two-dimensional (2D) condensates, major insights have been achieved through the controlled generation and manipulation of vortex–antivortex pairs using phase imprinting (16–18) or the so-called “chopsticks” method (19). In nonsimply connected geometries, vortex dynamics sustains quantized persistent currents (20), underpinning key developments in the emerging field of atomtronics (21, 22). The dynamics of 3D vortices, however, poses an additional level of complexity. Dissipation (23), the interplay between vortex tangles and lines (24), and reconnections (25–27) represent formidable challenges for both real-time numerical simulations and experiments (28–33). Establishing a controlled platform to generate and analyze 3D vortex dynamics is therefore of great importance.

**Fig. 1.**
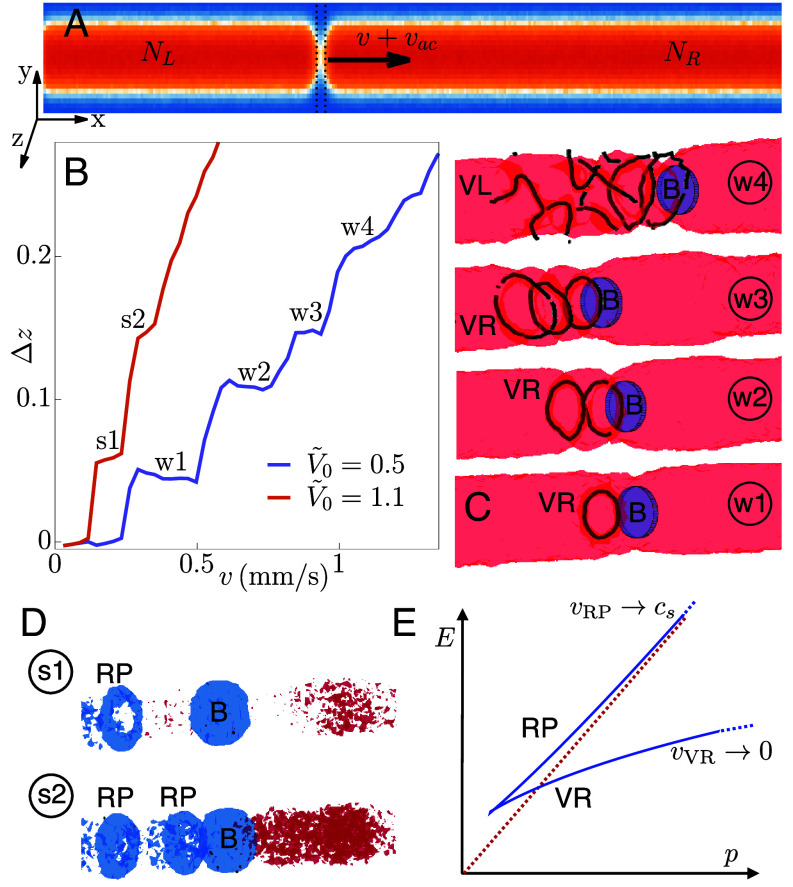
On-demand generation of Jones-Roberts excitations in trapped 3D BEC. (*A*) Simulation of the atomic Josephson junction (AJJ), which is created by separating two 3D clouds with a Gaussian barrier of height $V_{0}$ and width w (indicated by two vertical dotted lines). $N_{L}$ ($N_{R}$) represents the atom number of the *Left* (*Right*) reservoir. We use w=1.1μm, and ${\tilde{V}}_{0}\equiv V_{0}/\mu$ in the range 0.4 to 1.65, where μ is the transverse trap-averaged chemical potential. dc and ac drives are implemented via the barrier position $x\left(t\right)=vt+x_{1}\text{sin}\left(2\pi ft\right)$, where v is the dc velocity, f is the ac frequency, and $x_{1}$ is the ac amplitude related with the ac velocity $v_{\mathrm{ac}}=2\pi fx_{1}$. (*B*) Velocity-imbalance (v−Δz) characteristics for ${\tilde{V}}_{0}=0.5$ and 1.1. (*C*) Semitransparent isosurface $n\left(r,t\right)/n_{0}=0.2$ shows the emission of one, two, three, and four vortex rings (VRs) at first, second, third, and fourth Shapiro steps, respectively, for ${\tilde{V}}_{0}=0.5$. The oscillating barrier (blue disc, labeled *B*) emits both VRs and phonons per drive cycle. Extracted vortex polylines overlaid as black tubes; closed loops correspond to VRs and open curves to vortex lines (VLs). (*D*) At ${\tilde{V}}_{0}=1.1$, the barrier nucleates rarefaction pulses (RPs), visible as hallow reduced density shells in the background subtracted isosurfaces. One and two RPs per drive cycle occur at first and second Shapiro steps, respectively. The density ripples (reduction as blue and increase as red) depict phonons. Full propagation dynamics of VRs and RPs are provided as movies in *SI Appendix*. (*E*) Sketch of the energy–momentum spectrum of the JR family of 3D solitary waves (34, 35). Beyond the finite-momentum cusp, the two branches represent VR and RP excitations. The dashed line $E=c_{s}p$ denotes the phononic spectrum, where $c_{s}$ is the sound velocity.

A cornerstone in this context is the family of Jones-Roberts solitary-wave solutions of the 3D Gross–Pitaevskii equation (GPE) (Fig. 1*E*). Using a combination of numerical and analytical methods, Jones and Roberts identified a continuous branch of solutions that smoothly connects quantized vortex rings (VRs) to vorticity-free rarefaction pulses (RPs) (34, 35). In real dynamics, these excitations manifest as transient structures that decay through sound emission, vortex reconnections, or Kelvin-wave cascades. The low-velocity branch corresponds to VRs carrying quantized circulation. As the momentum decreases, the ring velocity increases up to a finite energy–momentum cusp, beyond which the branch connects continuously to RPs propagating near the sound velocity. This unified family thus reveals a natural dynamical pathway by which a VR can shrink, shed its vorticity at the cusp, and transform into an RP-like excitation.

While specific features of these solitonic excitations have been explored theoretically (35–42) and experimentally (43–46), a systematic experimental probe of the full JR family—from vortex rings to rarefaction pulses—has remained elusive. Previous theoretical studies have shown that VRs interact via Biot–Savart-like induction: Generic, noncoaxial collisions excite Kelvin waves along the filaments, leading to sound emission and eventual ring breakup (47–53). For coaxial configurations, vortex rings exhibit “leapfrogging” dynamics, alternately decelerating and accelerating as they exchange momentum.

Experiments in superfluid helium have visualized rings, reconnections, and sound-mediated decay, thereby anchoring these mechanisms experimentally (54–56). In particular, generalized leapfrogging dynamics of vortex rings was discussed using Euler-fluid description (57). In trapped atomic condensates, finite temperature and spatial inhomogeneity introduce additional damping (mutual friction) that accelerates shrinkage and decay (58–60).

Here, we propose driven atomic Josephson junctions (AJJs) as a tunable and reproducible platform for generating JR excitations on demand and mapping their dynamics. Relying on Shapiro steps, we demonstrate that by tuning the barrier strength and drive amplitude, each drive cycle can be engineered to produce either VRs or RPs. We then i) extract ring radii and velocities from 3D isosurfaces and vortex-filament reconstructions, ii) analyze the column-density deficit as a proxy for interaction energy, and iii) decompose the kinetic energy into compressible and incompressible components to track energy exchange between vortices and phonons. Together, these results reveal the VR-RP crossover, delineate the parameter space where it occurs, and clarify how compressibility, confinement, and finite temperature govern excitation lifetimes and decay pathways. Beyond establishing a clean 3D fingerprint of the JR family, our findings provide a quantitative benchmark for nonequilibrium vortex-phonon dynamics in quantum fluids and open a pathway toward studying device-relevant dissipative mechanisms. Finally, we demonstrate that our protocol enables controlled studies of ring–ring interactions and leapfrogging dynamics. All results are obtained from finite-temperature classical-field simulations.

## Simulation Method

We consider a weakly interacting Bose–Einstein condensate (BEC) of ^87^Rb atoms confined in a 3D tube-like geometry interrupted by a narrow, movable optical barrier that forms an AJJ (60, 61). The barrier is driven by combined dc and ac motions to induce Shapiro steps (61–63). We simulate the dynamics using a classical-field method within the truncated Wigner approximation (64–67). The system is described by the Hamiltonian

[1] $$\begin{array}{cc} {\hat{H}}_{0} & =\int \text{d}r[\frac{\hbar^{2}}{2m}\nabla {\hat{\psi }}^{†}\left(r\right)\cdot \nabla \hat{\psi }\left(r\right)+V_{\text{h}}\left(r\right){\hat{\psi }}^{†}\left(r\right)\hat{\psi }\left(r\right)\;\\ & +\frac{g}{2}{\hat{\psi }}^{†}\left(r\right){\hat{\psi }}^{†}\left(r\right)\hat{\psi }\left(r\right)\hat{\psi }\left(r\right)], \end{array}$$

$\hat{\psi }$ (${\hat{\psi }}^{†}$) is the bosonic annihilation (creation) operator. The 3D interaction parameter is given by $g=4\pi a_{s}\hbar^{2}/m$, where $a_{s}$ is the s-wave scattering length and m is the mass. We employ the parameters corresponding to the ^87^Rb platform used in recent experiments (60, 61), where $a_{s}=5.3\;\text{nm}$. The external confinement is a harmonic potential $V_{\text{h}}\left(r\right)=m\left(\omega_{x}^{2}x^{2}+\omega_{y}^{2}y^{2}+\omega_{z}^{2}z^{2}\right)/2$ with frequencies $\left(\omega_{x},\;\omega_{y},\;\omega_{z}\right)=2\pi \times \left(1.6,\;252,\;250\right)\;\text{Hz}$. Within the classical-field approximation, we replace the operators $\hat{\psi }$ in Eq. **1** and in the equations of motion by complex numbers ψ. We map real space on a lattice system of 320×25×25 sites with the discretization length of 0.25μm. We sample the initial states ψ(r,t=0) from a grand canonical ensemble at temperature T and chemical potential $\mu_{0}$ via a classical Metropolis algorithm. To obtain clear snapshots and extended lifetimes of the solitary waves, the temperature is set to T=2.2nK. At higher temperatures, the qualitative behavior persists, but the excitations decay faster (60, 61). We adjust $\mu_{0}$ so that the total atom number is N≃165,000, yielding a maximum density $n_{0}={208\;\mu \text{m}}^{-3}$ at the trap center. Relative to the critical temperature $T_{c}$ of an ideal trapped Bose gas, this corresponds to $T/T_{c}∼0.01$, indicating that the system is deeply in the degenerate regime. The resulting ensemble therefore incorporates thermal and quantum fluctuations within the classical-field dynamics description. We note that the deterministic excitation generation discussed here is expected to persist within the zero-temperature Gross–Pitaevskii equation (GPE) framework. Finite-temperature extensions such as stochastic-GPE and Zaremba–Nikuni–Griffin approaches may additionally capture dissipative coupling to thermal excitations and reservoir dynamics (64, 68), thereby providing a complementary description to the present approach.

We propagate each initial state using the equations of motion

[2] $$\begin{array}{c} i\hbar \dot{\psi }\left(r,t\right)=(-\frac{\hbar^{2}}{2m}\nabla^{2}+V_{\text{h}}\left(r,t\right)+V_{\text{b}}\left(r,t\right)+g{\left|\psi \right|}^{2})\psi \left(r,t\right), \end{array}$$

which include the barrier potential of the form

[3] $$\begin{array}{c} V_{\text{b}}\left(r,t\right)=V_{0}\left(t\right)\exp[-\frac{2(x-x_{0}-x\left(t\right){)}^{2}}{w^{2}}], \end{array}$$

with width w=1.1μm and center $x_{0}=28\;\mu \text{m}$. $V_{0}\left(t\right)$ is the barrier’s strength and x(t) is the time-dependent location. We first ramp $V_{0}$ linearly to its desired value over 200ms and wait for 50ms to equilibrate. This effectively separates the condensate into two subclouds, thus creating an AJJ by suppressing density at location $x_{0}$, see Fig. 1*A*. We then drive the barrier using $x\left(t\right)=vt+x_{1}\text{sin}\left(2\pi ft\right)$, where v is the dc velocity, $x_{1}$ is the ac amplitude, and f is the ac frequency (62). This induces phase locking between the junction dynamics and the external drive, leading to deterministic phase slips at the junction during each drive cycle (63). We choose f=90Hz such that the resulting Shapiro steps are well resolved (61). We vary $x_{1}$ in the range 0.125−1μm and fix the driving time to 3 cycles. We use the transverse trap-averaged chemical potential $\mu =2\mu_{0}/3$, where $\mu_{0}$ is the chemical potential at the trap center (60). We define the normalized barrier height by ${\tilde{V}}_{0}\equiv V_{0}/\mu$, and vary ${\tilde{V}}_{0}$ in the range 0.4 to 1.65.

We calculate the local density $n\left(r,t\right)={\left|\psi \left(r,t\right)\right|}^{2}$ and the column density n(x,t)=∫dydzn(r,t), and express time in units of the drive period $T_{f}=1/f=11.11\;\text{ms}$. The value of the sound velocity, determined from the propagation of a phonon pulse in the column density, is $c_{s}=1.83\;\text{mm/s}$. For velocity-imbalance characteristics, we quantify the imbalance $\Delta z=z-z_{\text{ref}}$ from atom numbers in the left and right reservoir, where $z_{\text{ref}}$ is taken from a reference cloud without the barrier. For vortex identification, we employ a plaquette-based phase-circulation criterion and reconstruct 3D vortex filaments to distinguish vortex rings and lines (see Materials and Methods).

To analyze energy exchange between vortex and sound channels, we decompose the total kinetic energy into compressible ($E_{k}^{c}$) and incompressible ($E_{k}^{i}$) components (69–72). We first compute the density-weighted velocity field $w\left(r\right)=j\left(r\right)/\sqrt{n(r)}$, where $j=\left(\hbar /m\right)I\left(\psi^{*}\nabla \psi \right)$. The Helmholtz decomposition is performed spectrally by projecting the Fourier-transformed velocity field onto its longitudinal $w_{\left|\right|}$ and transverse $w_{⊥}$ parts, with $\nabla \times w_{\left|\right|}=0$ and $\nabla \cdot w_{⊥}=0$, encoding phonons and vorticity channels, respectively. The corresponding energy densities are

[4] $$\begin{array}{c} E_{k}^{c}=\frac{1}{2}\int |w_{\left|\right|}{|}^{2}dr,\;E_{k}^{i}=\frac{1}{2}\int {\left|w_{⊥}\right|}^{2}dr. \end{array}$$

The resulting $E_{k}^{c}\left(t\right)$ and $E_{k}^{i}\left(t\right)$ traces are expressed in units of nanoKelvin per atom after subtracting the baseline value averaged over the initial equilibrium window. This approach cleanly separates vortex-associated (incompressible) and phonon-associated (compressible) motion, allowing direct tracking of vortex-phonon energy conversion during creation and decay dynamics.

## Results

We first analyze phase locking between the junction and the ac drive via the imbalance response after three drive cycles. The v−Δz characteristics exhibit clear Shapiro steps for both weak (${\tilde{V}}_{0}=0.5$) and strong (${\tilde{V}}_{0}=1.1$) barriers, confirming synchronization over a broad range, see Fig. 1*B*. For weak barriers, steps are sharply resolved, while for strong barriers the first two remain visible. The corresponding critical velocities without ac drive are $v_{c}\approx 0.58$ and 0.16mm/s, respectively. Each n-th step corresponds to the deterministic emission of n nonlinear excitations per cycle. At ${\tilde{V}}_{0}=0.5$, one, two, three, and four coaxial VRs are emitted per cycle at the first, second, third, and fourth steps, respectively (Fig. 1*C*). The oscillating barrier injects energy near the junction, shedding VRs and phonons whenever the instantaneous barrier velocity exceeds the local critical velocity.

At higher barrier heights, the local density in the junction decreases, thereby increasing the local healing length. Under these conditions, it becomes difficult for the system to sustain well-defined topological vortex-core excitations, and the generated nonlinear excitations shift from VRs to RPs (Fig. 1 *D* and *E*). For ${\tilde{V}}_{0}=1.1$, the first and second steps yield one and two RPs per cycle, respectively, while phonon emission appears as red or blue density ripples. This VR-RP crossover parallels the weak-link to tunneling crossover in dc-driven AJJs (60).

In summary, we find that i) the Shapiro step index determines the number of nonlinear excitations emitted per cycle, ii) the barrier height selects the excitation type (VR or RP), and iii) our phase-circulation-based filament analysis enables unambiguous classification and dynamical tracking of each excitation.

### Excitations at First Shapiro Step.

**Fig. 2.**
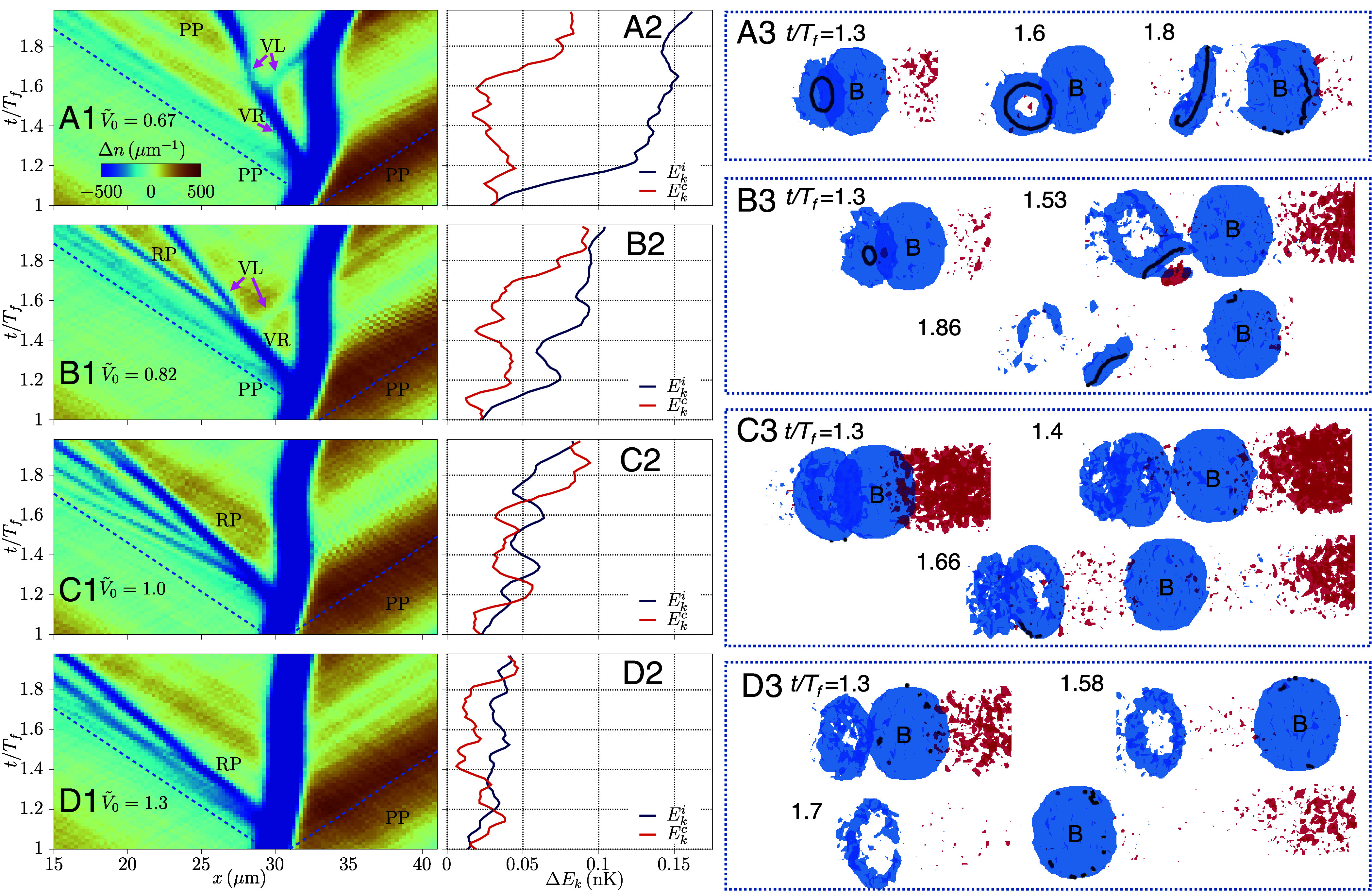
Dynamics of JR excitations at the first Shapiro step. (*A1*–*D1*) Time evolution of the column density $\Delta n\left(x,t\right)=n\left(x,t\right)-n_{0}\left(x\right)$ at different barrier heights ${\tilde{V}}_{0}$, where $n_{0}\left(x\right)$ is the density without the barrier. Time is given in units of the drive period $T_{f}$. The oscillating barrier (thick depletion) generates both phononic (linear) and nonlinear excitations, with the latter propagating below the sound velocity. Density pulses include vortex ring (VR), vortex line (VL), rarefaction pulse (RP), and phononic pulse (PP). The dashed lines represent the slope of speed of sound $c_{s}=1.83\;\text{mm/s}$. (*A2*–*D2*) Corresponding compressible $E_{k}^{c}$ and incompressible $E_{k}^{i}$ kinetic energy components. (*A3*) For ${\tilde{V}}_{0}=0.67$, the isosurface $\Delta n\left(r,t\right)/n_{0}=0.2$ shows nucleation of a VR (closed polyline) and phonons (density excess in red) at $t/T_{f}=1.3$. During evolution, the VR bends toward the transverse boundary and generally decays into two VLs (open loops) propagating in opposite directions. B marks the density reduction at the barrier. (*B3*) For ${\tilde{V}}_{0}=0.82$, a small-radius VR is nucleated, which dissipates its energy and momentum into an RP (hollow ring) and a VL (open polyline). Their different characteristic velocities allow them to separate over time. (*C3* and *D3*) At ${\tilde{V}}_{0}=1$ and 1.3, the barrier mainly emits an RP (hollow ring) and phonons.

At the first Shapiro step, the oscillating barrier injects energy periodically and drives the local flow above the critical velocity once per cycle. This produces a reproducible sequence of excitations in each drive period: fast phonon pulses propagating near the sound velocity and a slower nonlinear density dip whose character depends on the barrier height, see Fig. 2. The time evolution of the column density Δn(x,t) makes this separation of velocities explicit and enables tracking of the dominant nonlinear excitation from its nucleation at the barrier into the bulk of the condensate.

From 3D isosurfaces and vorticity analysis, we identify two regimes. For moderate barrier heights (${\tilde{V}}_{0}\approx 0.4$ to 0.8), a VR is created, propagates through the condensate, and eventually decays. For higher barriers (${\tilde{V}}_{0}≳1$), the dominant nonlinear excitation is a RP without phase singularity. The column-density evolution reveals broad phonon pulses moving at nearly the sound velocity and a slower, more localized dip corresponding to the nonlinear excitation. The 3D snapshots confirm the topology—closed vortex loops for VRs versus hollow density shells without circulation for RPs. We note that the numerical precision is sufficient to resolve vortex cores and associated phase singularities on the relevant microscopic scale. In the RP regime, at later times, secondary short vortex-line segments may appear around the hollow density structures due to deformation, sound emission, and interactions with the inhomogeneous background. These later defects are distinct from an unresolved primary VR core and do not change the identification of the emitted excitation as RP-like. Cycle-resolved kinetic energy decomposed into incompressible $E_{k}^{i}$ and compressible $E_{k}^{c}$ components clarifies the underlying energy exchange. When a VR is created, $E_{k}^{i}$ rises sharply while $E_{k}^{c}$ increases only weakly, indicating most of the injected energy initially feeds vorticity. As the ring decays, energy transfers from $E_{k}^{i}$ to $E_{k}^{c}$, capturing the sound emission accompanying breakup. In the RP regime, both $E_{k}^{c}$ and $E_{k}^{i}$ exhibit weak, transient oscillations with no dominant component—consistent with the absence of quantized circulation and the weak propagation of the RP. During evolution, the RP continuously radiates energy into sound, visible as small density clouds detaching from the main pulse. This gradual energy leakage marks the conversion of the localized excitation into dispersive phonons, reflecting its coupling to the continuum of sound modes. The RP regime thus represents the smooth, high-velocity end of the JR family, where vortex-free RPs continuously merge into the phonon branch (35).

The decay pathways are governed by confinement and background inhomogeneity. In the VR regime, image-flow attraction and background inhomogeneity tilt the ring toward the transverse boundary; contact with the edge breaks the loop into two dominant vortex lines (VLs), one continuing in the ring’s direction and one reflecting toward the barrier. This event is accompanied by a clear transfer of energy from $E_{k}^{i}$ to $E_{k}^{c}$, signaling sound emission. This boundary-assisted breakup is the dominant decay channel for barriers up to ${\tilde{V}}_{0}∼0.7$ (Fig. 2*A1*–*A3*). For intermediate barriers, smaller ring radii and stronger damping lead to mixed decay channels producing both an RP and a residual VL (Fig. 2*B1*–*B3*). At higher barriers, the junction operates fully in the RP regime, and no topological breakup occurs; the resulting excitation disperses gradually while continuously carrying momentum away from the junction (Fig. 2*C1*–*D3*). We emphasize that internal ring-core excitations (Kelvin modes) remain weak during the early-time dynamics, where the VR maintains approximate rotational symmetry around its propagation axis. At later times, however, transverse confinement and interactions with the finite condensate boundary progressively amplify bending deformations, ultimately contributing to the instability and decay of the VR.

**Fig. 3.**
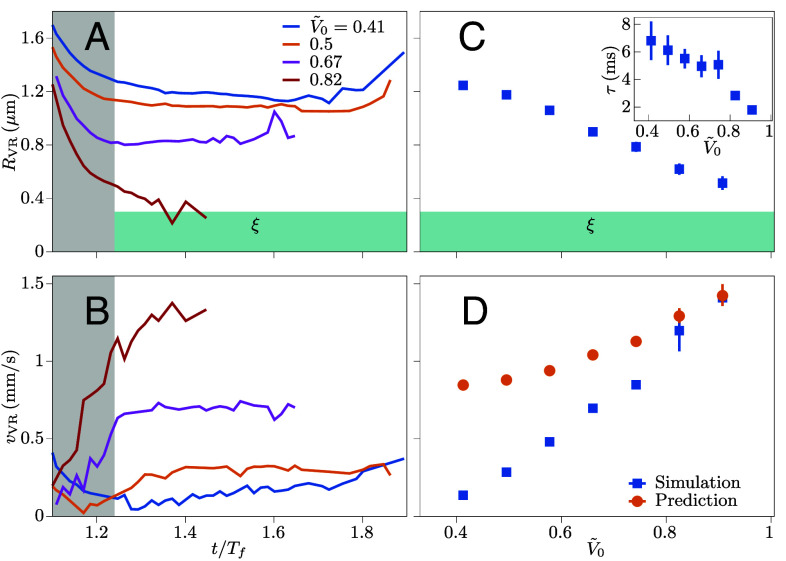
Characterization of single vortex rings. (*A* and *B*) Time evolution of the VR radius $R_{\text{VR}}\left(t\right)$ and velocity $v_{\text{VR}}\left(t\right)$ for a single sample in the ensemble at different ${\tilde{V}}_{0}$. The healing length ξ≃0.3μm (horizontal shaded region) defines the lower bound of $R_{\text{VR}}$, close to which the ring becomes unstable as $R_{\text{VR}}$ approaches the core radius (∼ξ). The vertical shaded region indicates the initial build-up time near the barrier. (*C* and *D*) Averaged $R_{\text{VR}}$ and $v_{\text{VR}}$ as a function of ${\tilde{V}}_{0}$. The *Inset* shows the corresponding ring lifetime τ, averaged over four samples; error bars represent the SD. In (*D*), circles represent predictions of the ring velocity $v_{\text{ring}}$ from Eq. **5**, using $R_{\text{VR}}$ values from panel (*C*); see text.

We next analyze the geometry and kinematics of the emitted VR. The time evolution of the ring radius $R_{\text{VR}}\left(t\right)$ and velocity $v_{\text{VR}}\left(t\right)$ for different ${\tilde{V}}_{0}$ is shown in Fig. 3 *A* and *B*. The rapid early-time decrease of $R_{\text{VR}}$ reflects circulation accumulation near the barrier before the formation of a well-defined core. Once formed, both $R_{\text{VR}}$ and $v_{\text{VR}}$ reach a quasi-steady plateau prior to decay. As the barrier height increases, the steady-state $R_{\text{VR}}$ decreases while $v_{\text{VR}}$ increases—consistent with the JR family of low-velocity solitary waves, in which smaller rings travel faster. This behavior agrees with the analytical expression for a thin-core VR in a uniform condensate (73),

[5] $$\begin{array}{c} v_{\text{ring}}=\frac{\kappa }{4\pi R}[\ln(\frac{8R}{a})-0.615], \end{array}$$

where κ=h/m is the circulation quantum and a≃ξ is the core radius. Using simulated $R_{\text{VR}}$ as input yields reasonable agreement with observed velocities, especially at higher ${\tilde{V}}_{0}$, where boundary effects are minimal and the motion approaches the uniform-space prediction (Fig. 3*D*). We note that increasing the transverse condensate size would allow the generation and stable propagation of larger VRs at lower barrier heights. This emphasizes that the characteristic VR size observed in the present setup is primarily determined by the finite transverse confinement and junction geometry, rather than by an intrinsic limitation of the generation protocol itself.

**Fig. 4.**
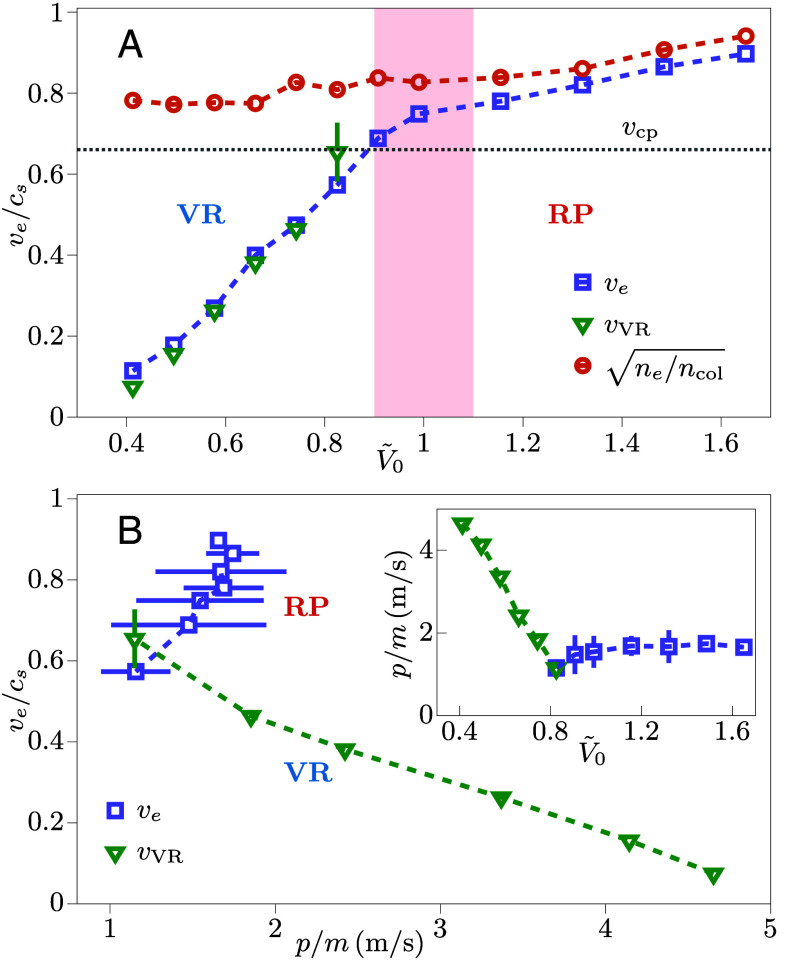
Vortex-ring (VR) to rarefaction-pulse (RP) crossover. (*A*) Solitary-wave velocity $v_{e}$ (squares), normalized by the sound velocity $c_{s}$, as a function of ${\tilde{V}}_{0}$. The crossover between VR and RP excitations occurs near ${\tilde{V}}_{0}∼1$ (vertical red shaded region). Triangles show the VR velocity $v_{\text{VR}}$ from Fig. 3*D*. The Bogoliubov estimate of the sound velocity, $v_{\text{B}}/c_{s}=\sqrt{n_{e}/n_{\text{col}}}$ (circles), is based on the reduced density $n_{e}$ at the dip location, where $n_{\text{col}}$ is the column density without the excitation. The horizontal dashed line at $v_{\text{cp}}/c_{s}\approx 0.66$ marks the minimum of the energy–momentum dispersion (the cusp) of GPE solutions, where the low-velocity (VR) and high-velocity (RP) branches merge (35). (*B*) $v_{e}$ and $v_{\text{VR}}$ as a function of momentum p. The *Inset* shows the VR (triangles) and RP (squares) momenta at varying ${\tilde{V}}_{0}$; see text. Error bars in momentum indicate the uncertainty arising from atom-number estimation within the pulse.

To unify the VR-RP crossover, we extract the solitary-wave velocity $v_{e}$ of the dominant nonlinear dip from Δn(x,t) and compare it with both the tracked ring velocity $v_{\text{VR}}$ and a local Bogoliubov estimate $v_{\text{B}}/c_{s}=\sqrt{n_{e}/n_{\text{col}}}$, based on the reduced density $n_{e}$ at the dip center and the density $n_{\text{col}}$ without the excitation (Fig. 4*A*). For ${\tilde{V}}_{0}≲0.9$, $v_{e}$ coincides with $v_{\text{VR}}$ and follows the decreasing-radius, increasing-velocity trend described above. Around ${\tilde{V}}_{0}∼1$, the behavior changes smoothly: $v_{e}$ increases only weakly and converges toward the RP branch identified from the isosurfaces. This marks the transition from vortex-carrying solitary waves to vorticity-free RPs. Physically, stronger barriers inject shorter-wavelength, more compressive perturbations that fail to sustain a quantized core; energy and geometry then favor a dispersive soliton-like excitation over a topological defect.

For a circular (thin-core) VR, the momentum is $p_{\text{VR}}=\pi n_{0}m\kappa R^{2}$, predicting a quadratic dependence on R (73). Using simulated $R_{\text{VR}}$ as input yields $p_{\text{VR}}$. For RPs, the total momentum along x is obtained from the continuity equation, $p_{x}\approx mv_{\text{RP}}\Delta N$, where ΔN is the atom number reduction within the pulse relative to the unperturbed cloud. Computing ΔN gives $p_{\text{RP}}$. Both quantities are shown in the *Inset* of Fig. 4*B*. While $p_{\text{RP}}$ varies weakly with ${\tilde{V}}_{0}$, $p_{\text{VR}}$ increases rapidly with decreasing ${\tilde{V}}_{0}$. In Fig. 4*B*, $v_{e}$ and $v_{\text{VR}}$ are plotted versus total momentum p, revealing two branches consistent with the JR dispersion (34): The VR branch exhibits increasing velocity with decreasing momentum up to a cusp, beyond which the RP branch emerges with increasing velocity approaching the sound velocity.

Together, these results show that i) the junction at the first Shapiro step emits one well-defined nonlinear carrier per cycle; ii) the carrier evolves continuously from a quantized vortex ring to a vorticity-free rarefaction pulse as $V_{0}$ increases; and iii) the partition of kinetic energy into incompressible and compressible components provides a direct dynamical fingerprint of the excitation’s topology.

### Dynamics at Higher Steps.

**Fig. 5.**
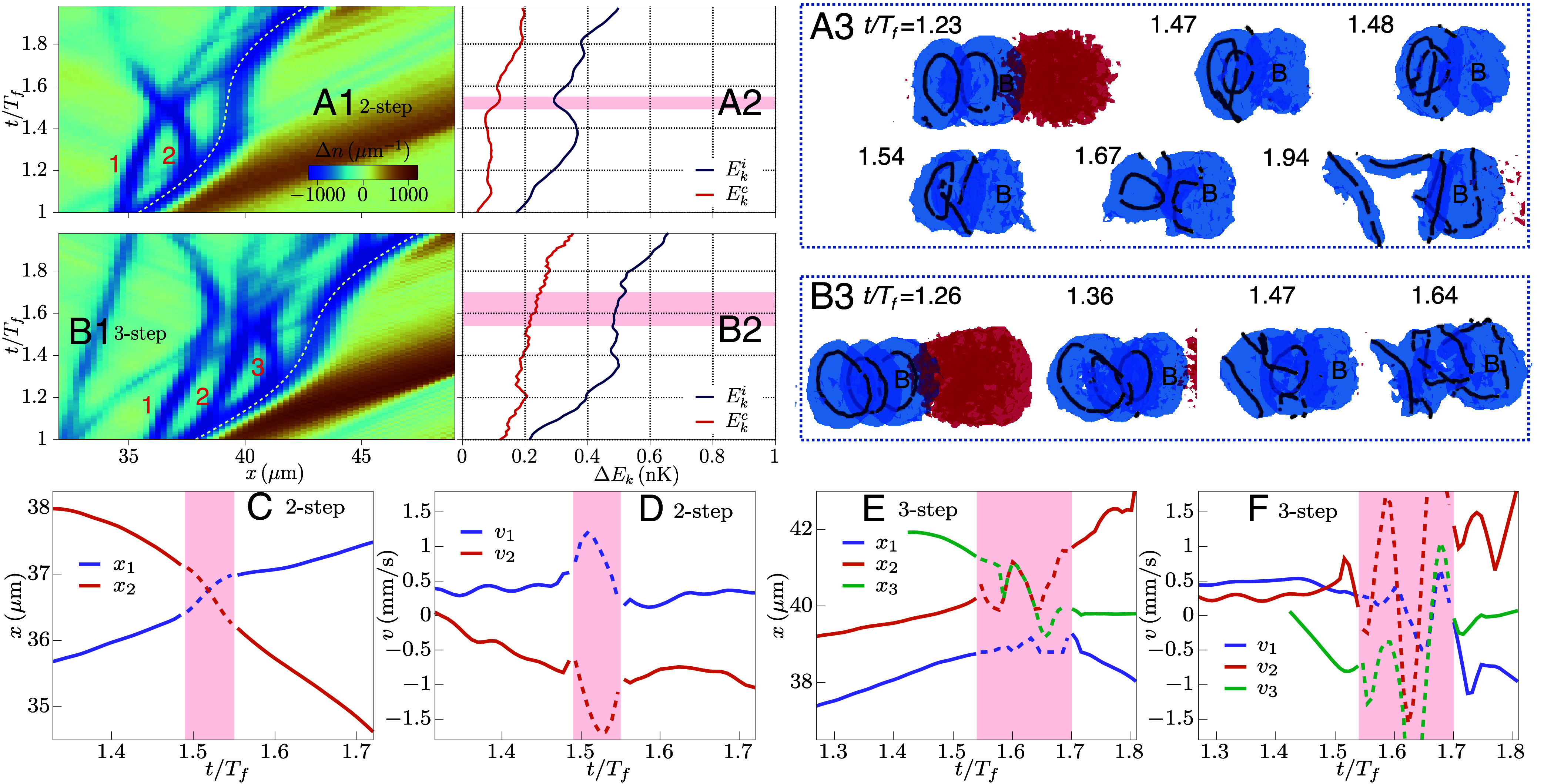
Leapfrogging dynamics at higher Shapiro steps for ${\tilde{V}}_{0}=0.5$. (*A1* and *B1*) Time evolution of the column density Δn(x,t) between cycles 1 and 2, showing the nucleation of two and three VRs at the second and third Shapiro steps, respectively. The barrier location is indicated as the dashed line. When the velocity fields of neighboring VRs overlap, they exhibit leapfrogging dynamics, leading to intricate dynamics for systems with more than two rings. (*A2* and *B2*) Corresponding compressible $E_{k}^{c}$ and incompressible $E_{k}^{i}$ components of the kinetic energy per particle. (*A3*) Isosurface snapshots of the density difference showing leapfrogging between two sequentially created VRs, which subsequently decay into vortex lines (open loops). (*B3*) Sequential creation and interaction of three VRs at the third Shapiro step. (*C*–*F*) Time evolution of the positions and velocities of the dominant nonlinear density dips at second and third steps, determined after averaging Δn(x,t) over 32 samples. The shaded region and dotted lines display the scattering time window, where two (*C* and *D*) and three (*E* and *F*) VRs interact and their subsequent decay dynamics follows. Small amplitude oscillations of solid lines and large amplitude oscillations of dashed lines are artifacts of numerical noise and derivatives.

We now exploit the ability to repro-ducibly nucleate VRs to probe their nonlinear dynamics at higher Shapiro steps. Throughout this section, the barrier height is fixed at ${\tilde{V}}_{0}=0.5$, and we analyze the behavior at the second and third steps in Fig. 5.

At the second Shapiro step, the barrier ejects two like-signed, coaxial VRs in sequence. As the rings approach one another, their velocity fields overlap and induce axial flows via Biot–Savart-like induction. This mutual coupling generates opposite radial strains: The leading ring expands while the trailing ring compresses. Because the self-induced velocity of a ring decreases with radius, the leading ring slows and the trailing ring accelerates, resulting in the characteristic exchange-of-position or “leapfrogging” motion. In an ideal, unbounded, incompressible fluid, this dynamics would be nearly periodic (48, 49, 58, 74). In our trapped, compressible superfluid, however, the leapfrogging dynamics are strongly affected by compressibility and confinement. Although the rings initially undergo the characteristic exchange of position, sound emission, ring deformation, and boundary interactions rapidly destabilize the configuration, ultimately leading to breakup into vortex lines. The associated decay and sound radiation appear as coincident drops in $E_{k}^{i}$ and spikes in $E_{k}^{c}$. While a detailed analysis of internal-core excitations is beyond the scope of the present work, indications emerge that Kelvin-mode oscillations may be excited during the multiring interactions. Transverse confinement further destabilizes the expanded (slower) ring; upon reaching the Thomas–Fermi (TF) boundary, the ring fragments into vortex lines, followed by the decay of the trailing ring.

At the third Shapiro step, three like-signed rings are emitted sequentially (Fig. 5*B1*–*B3*). The first two initially display the same leapfrogging behavior, but the arrival of the third ring reshapes the induction landscape: Its flow slows and expands the second ring, counteracting the compression caused by the first. The resulting push–pull competition frustrates clean two-body leapfrogging: Three-body induction drives the leading rings toward the TF boundary, where both destabilize and break into VLs. The resulting mixture of vortex lines and emitted phonons perturbs the third ring, which briefly accelerates before decaying. Corresponding energy traces again show bursts in $E_{k}^{c}$ synchronized with declines in $E_{k}^{i}$, indicating repeated vortex-to-sound energy transfer during the cascade.

To quantify these dynamics, we track the positions of the dominant nonlinear density dips from the column-density evolution Δn(x,t), averaged over 32 samples to suppress background and residual excitation effects. Time derivative of these positions yields the instantaneous velocities. The corresponding trajectories and velocities for the second and third Shapiro steps are shown in Fig. 5*C*–*F*. In the two-ring case (second step), the locations $x_{1}\left(t\right)$ and $x_{2}\left(t\right)$ exhibit alternating acceleration and deceleration within a well-defined scattering window (shaded region), marking successive leapfrogging events. The associated velocities $v_{1}\left(t\right)$ and $v_{2}\left(t\right)$ are out of phase, confirming reciprocal induction between the two rings: Each expansion of the leading ring coincides with compression and acceleration of the trailing one. In the three-ring case (third step), the trajectories $x_{1,2,3}\left(t\right)$ display more complex coupling. Initially, the first two rings undergo partial leapfrogging, but as the third ring enters the interaction region, mutual three-body induction alters the phase relation—manifesting as irregular velocity changes and enhanced damping. The scattering window delineates the period during which the three rings overlap and exchange momentum before decaying into VL fragments. Overall, this quantitative analysis demonstrates that energy and momentum transfer during multiring collisions proceeds through repeated, inelastic leapfrogging cycles.

## Discussion and Outlook

A driven atomic Josephson junction provides a compact and tunable platform to generate and study the complete Jones-Roberts (JR) family of solitary waves in three dimensions. By tuning the barrier height and drive amplitude, the system continuously spans the spectrum from quantized vortex rings (VRs) to vorticity-free rarefaction pulses (RPs). The Shapiro-step phenomenon offers direct control over the number of emitted excitations and their interaction: The first step generates a single nonlinear excitation, while higher Shapiro steps produce multiple excitations per cycle. This precise control enables reproducible access to distinct dynamical regimes and establishes a route for deterministic generation of topological and nontopological solitary waves in superfluids.

The combined analysis of geometry, kinematics, and energetics, reveal how these excitations exchange energy with sound, clarifying the microscopic origin of phase-slip dissipation and vortex-phonon coupling in driven junctions. Shapiro phase locking allows emission of close by excitations, enabling controlled studies of leapfrogging dynamics, sound-mediated decay, and vortex-line formation—key processes of superfluid turbulence and atomtronic transport. The platform can also be extended toward more bulk-like regimes by tuning the trap geometry, atom number, and junction parameters such that boundary-induced effects are substantially suppressed. This would provide access to vortex-ring dynamics closer to the unbounded three-dimensional limit.

Future extensions using in-situ imaging could directly visualize the emission and decay of these nonlinear excitations. Such measurements can be realized using selective imaging techniques (75, 76) capable of reconstructing entire 3D condensate profiles slice by slice. Extending the present protocol to multicomponent condensates could further enable the study of hybrid vortex-soliton structures, intercomponent drag effects (32, 77), and vortices with massive cores (78, 79). These directions will broaden the accessible landscape of nonlinear excitations and advance the design of superfluid quantum circuits.

After the completion and submission of this work, Iori et al. (80) reported the reproducible nucleation and control of vortex rings in a dc-driven atomic Josephson junction. Unlike the present work, their study did not address Shapiro-step phase locking or the relation of the emitted excitations to the Jones–Roberts spectrum of 3D solitary waves.

## Materials and Methods

### Calculation of Vortex Filaments.

We extract vortex filaments from ψ(r,t) using a plaquette-phase criterion. For each grid face we compute the wrapped phase circulation $\Gamma_{□}=\sum_{j}^{4}\Delta \phi_{j}$, where $\Delta \phi_{j}=\phi_{j+1}-\phi_{j}\in \left(-\pi ,\pi \right]$. Faces with $|\Gamma_{□}|>\pi /2$ are tagged as pierced by a vortex. Core positions are then refined to subgrid accuracy by bilinear intersection of Rψ=0 and Iψ=0. Nearby intersection points are paired into short segments and merged with a small tolerance; segments are then assembled into continuous polylines by an adjacency graph walk. Closed polylines are classified as VRs and fitted to a best-fit plane and circle to obtain ring center, radius, and normal. Filaments are linked across time frames by nearest-neighbor matching of ring centers to yield trajectories and velocities. Open filaments are characterized as vortex lines (VLs). The analysis is performed within the Thomas-Fermi (TF) radii in yz directions to suppress boundary artifacts. This procedure follows established GPE-vortex extraction methods (e.g. refs. 81–84) and is numerically robust to thermal noise and finite resolution. For visualization we render the calculated polylines as tubes atop semitransparent density isosurfaces (Fig. 1*C*).

## Supplementary Material

Appendix 01 (PDF)

Movie S1. This movie shows the 3D density profile of the condensate as the barrier is driven with combined dc and ac motion in the dc velocity range corresponding to the first Shapiro step. The movie displays the time evolution over one drive cycle between the first and second cycles (see simulation parameters in Fig. 1 of the main text). Black tubes represent vortex filaments extracted from the complex wavefunction: closed loops correspond to vortex rings, while open curves indicate vortex lines crossing the condensate. A vortex ring nucleates at the junction, detaches, and propagates into the condensate.

Movie S2. This movie shows the time evolution in the dc velocity range corresponding to the second Shapiro step. Two vortex rings nucleate in sequence at the junction and propagate into the condensate while undergoing vortex–vortex interaction dynamics.

Movie S3. This movie corresponds to the third Shapiro step, where three vortex rings are nucleated sequentially at the junction. The resulting interaction dynamics is increasingly complex due to the presence of multiple excitations.

Movie S4. This movie shows the dynamics of the density difference with respect to a reference cloud taken in the absence of excitations. In the regime of a high barrier height—where vortex-ring excitations are suppressed and rarefaction pulses (RPs) dominate (see simulation parameters in Fig. 1 of the main text)—the time evolution for the dc velocity range corresponding to the first Shapiro step shows the nucleation of a single RP propagating through the condensate.

Movie S5. This movie shows the density-difference dynamics in the dc velocity range corresponding to the second Shapiro step, where two rarefaction pulses are generated in sequence at the barrier and propagate through the condensate.

The SI Appendix has been updated to correct an author affiliation.

## Data Availability

Study data are included in the article and/or supporting information.
