# Supplementary material for: Controlled generation of 3D vortices in driven atomic Josephson junctions
Source: Proc Natl Acad Sci U S A. 2026 Jul 7;123(28):e2535111123. doi: 10.1073/pnas.2535111123 (PMC13367877; doi:10.1073/pnas.2535111123)
Supplement: Supplementary file 1 — Appendix 01 (PDF) [file pnas.2535111123.sapp.pdf]

Supporting Information for

Controlled generation of 3D vortices in driven atomic Josephson junctions

Vijay Pal Singh[1], **Ludwig Mathey**[2], **Herwig Ott**[3], **and Luigi Amico**[4]

[1]Quantum Research Center, Technology Innovation Institute, Abu Dhabi, UAE; [2]Zentrum für Optische Quantentechnologien and Institut für Quantenphysik, Universität Hamburg, 22761 Hamburg, Germany; [2]The Hamburg Centre for Ultrafast Imaging, Luruper Chaussee 149, 22761 Hamburg, Germany; [3]Department of Physics and Research Center OPTIMAS, Rheinland-Pfälzische Technische Universität Kaiserslautern-Landau, 67663 Kaiserslautern, Germany; [4]Quantum Research Center, Technology Innovation Institute, Abu Dhabi, UAE

Vijay Pal Singh
E-mail: vijay.singh@tii.ae

This PDF file includes:

Legends for Movies S1 to S5

Other supporting materials for this manuscript include the following:

Movies S1 to S5

Movie S1. **This movie shows the 3D density profile of the condensate as the barrier is driven with combined dc and ac motion in the dc velocity range corresponding to the first Shapiro step. The movie displays the time evolution over one drive cycle between the first and second cycles (see simulation parameters in Fig. 1 of the main text). Black tubes represent vortex filaments extracted from the complex wavefunction: closed loops correspond to vortex rings, while open curves indicate vortex lines crossing the condensate. A vortex ring nucleates at the junction, detaches, and propagates into the condensate.**

Movie S2. **This movie shows the time evolution in the dc velocity range corresponding to the second Shapiro step. Two vortex rings nucleate in sequence at the junction and propagate into the condensate while undergoing vortex–vortex interaction dynamics.**

Movie S3. **This movie corresponds to the third Shapiro step, where three vortex rings are nucleated sequentially at the junction. The resulting interaction dynamics is increasingly complex due to the presence of multiple excitations.**

Movie S4. **This movie shows the dynamics of the density difference with respect to a reference cloud taken in the absence of excitations. In the regime of a high barrier height—where vortex-ring excitations are suppressed and rarefaction pulses (RPs) dominate (see simulation parameters in Fig. 1 of the main text)—the time evolution for the dc velocity range corresponding to the first Shapiro step shows the nucleation of a single RP propagating through the condensate.**

Movie S5. **This movie shows the density-difference dynamics in the dc velocity range corresponding to the second Shapiro step, where two rarefaction pulses are generated in sequence at the barrier and propagate through the condensate.**
